# Decoding the host–pathogen interspecies molecular crosstalk during oral candidiasis in humans: an *in silico* analysis

**DOI:** 10.3389/fgene.2023.1245445

**Published:** 2023-10-12

**Authors:** Ali Rejwan Kabir, Anis Ahmad Chaudhary, Malak O. Aladwani, Soumita Podder

**Affiliations:** ^1^ Computational and System Biology Lab, Department of Microbiology, Raiganj University, Raiganj, West Bengal, India; ^2^ Department of Biology, College of Science, Imam Mohammad Ibn Saud Islamic University (IMSIU), Riyadh, Saudi Arabia

**Keywords:** *Candida albicans*, oral candidiasis, protein–protein interaction network, interologs, biofilm, gene regulatory network, module

## Abstract

**Introduction:** The objective of this study is to investigate the interaction between *Candida albicans* and human proteins during oral candidiasis, with the aim of identifying pathways through which the pathogen subverts host cells.

**Methods:** A comprehensive list of interactions between human proteins and *C. albicans* was obtained from the Human Protein Interaction Database using specific screening criteria. Then, the genes that exhibit differential expression during oral candidiasis in *C. albicans* were mapped with the list of human–*Candida* interactions to identify the corresponding host proteins. The identified host proteins were further compared with proteins specific to the tongue, resulting in a final list of 99 host proteins implicated in oral candidiasis. The interactions between host proteins and *C. albicans* proteins were analyzed using the STRING database, enabling the construction of protein–protein interaction networks. Similarly, the gene regulatory network of *Candida* proteins was reconstructed using data from the PathoYeastract and STRING databases. Core module proteins within the targeted host protein–protein interaction network were identified using ModuLand, a Cytoscape plugin. The expression levels of the core module proteins under diseased conditions were assessed using data from the GSE169278 dataset. To gain insights into the functional characteristics of both host and pathogen proteins, ontology analysis was conducted using Enrichr and YeastEnrichr, respectively.

**Result:** The analysis revealed that three *Candida* proteins, HHT21, CYP5, and KAR2, interact with three core host proteins, namely, ING4 (in the DNMT1 module), SGTA, and TOR1A. These interactions potentially impair the immediate immune response of the host against the pathogen. Additionally, differential expression analysis of fungal proteins and their transcription factors in *Candida*-infected oral cell lines indicated that Rob1p, Tye7p, and Ume6p could be considered candidate transcription factors involved in instigating the pathogenesis of oral candidiasis during host infection.

**Conclusion:** Our study provides a molecular map of the host–pathogen interaction during oral candidiasis, along with potential targets for designing regimens to overcome oral candidiasis, particularly in immunocompromised individuals.

## Graphical Abstract

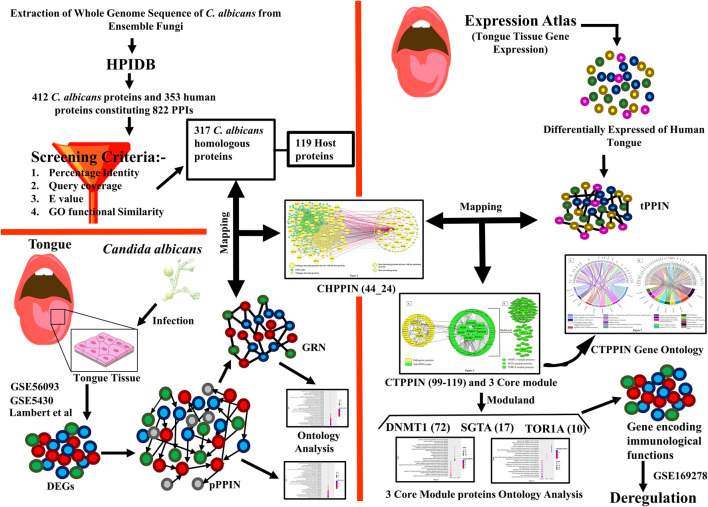

## 1 Introduction

For decades, the malady of oral candidiasis (OC) or thrush has been known to occur in people. Up to 95% of cases of OC are caused by *Candida albicans*, making it the primary causative agent of the disease (Vila et al., 2020). Though *Candida* is readily isolated from the oral cavity, its simple presence does not predictably result in the development of an infection as it could remain as a commensal or transmute into a pathogen. The chance of transition is usually determined by pre-existing or associated variations in the host immune system. *Candida* infections can range from superficial mucocutaneous illnesses to invasive, disseminated diseases affecting numerous organs. About 2 million people worldwide develop oral candidiasis every year (Dufresne et al., 2017). According to estimates, it affects 88% of AIDS patients (depending on the immunity status and geographic location) and nearly 20% of cancer patients (Lalla et al., 2013; Gaitán-Cepeda et al., 2015). In most cases, it has been observed that intraoral environmental changes or systemic variables, such as diabetes mellitus and immunodeficiency, are linked to the organism’s transition from commensalism to parasitism and ebullient growth (Patil et al., 2015). These predisposing conditions increase the infection probability by many folds. Basically, *C. albicans* is a dimorphic fungus, i.e., it can exist in both yeast and hyphal forms. In addition to hyphal form transition, biofilm development is now believed to play a crucial role in OC (Cho et al., 2021). The hyphal form predominates in pathogenic conditions associated with virulence, tissue damage, epithelial infiltration, keratinization, and biofilm formation (Rollenhagen et al., 2009; Richardson et al., 2018). Subsequent articles have affirmed that *Candida* pathogenicity in mucosal candidiasis depends on biofilm formation, which causes persistent infection, recurrence, and antifungal resistance compared to planktonic *Candida* (Cavalheiro and Teixeira, 2018; Wu et al., 2020). Since the mortality rate with systemic candidiasis is considerably higher (Akpan and Morgan, 2002; Pappas et al., 2018), it has enthralled clinicians to investigate its molecular mechanism of pathogenicity and to improvise newer therapeutic regimens based on the updated molecular research. *C. albicans* has the potential to thrive in different host niches and is considered one of the common residents of the epithelial mucosa of the oral cavity, airways, gastrointestinal tract, and genital tract (Dias, 2020; Liu et al., 2021; Patel, 2022).

Numerous research studies on oral candidiasis and several mutational studies on the key molecular players of *C. albicans* and its interactions with hosts disclosed unique immunological pathways effective for pathogen clearance. An *in vitro* study has shown that the ERG gene plays a pivotal role in triggering tissue damage that activates certain kinds of molecules such as c-Fos, mitogen-activated protein kinase 1 (MAPK1), and activating protein-1 (AP-1). Subsequently, mutation of the related genes Erg3 and Erg11 downregulates Als1, Als3, and Sap6, which facilitates escaping the macrophages and pathogenesis of *C. albicans* in the oral mucosa (Dalle et al., 2010; Hebecker et al., 2014; Zhou et al., 2018). Similarly, it has been reported that the mouse model of oropharyngeal candidiasis (OPC) has significantly reduced infectivity in double homozygous deletion mutants (tec1Δ/Δ, bcr1Δ/Δ, and rob1Δ/Δ) and one heterozygous mutant (tec1Δ/TEC1) (Solis et al., 2022). Additionally, the expression of the secreted aspartyl proteinase (SAP) molecule varies among different niches and was found to be in higher concentrations in oral candidiasis or vaginitis of HIV-infected patients (Bernardis et al., 1996). Moreover, during biofilm development during OC, the transcription factor Bcr1 plays a vital role, and adhesin proteins such as Hwp1 serves as an epithelial cell adhesin through the action of mammalian host transglutaminase, while Hyr1 is necessary for the switch from a mucosal surface biofilm to epithelial tissue invasion (Staab et al., 1999; Luo et al., 2010; Dwivedi et al., 2011).

Despite recent breakthroughs in our understanding, there are still many unanswered questions about the impact of these reported *Candida* genes on the molecular pathophysiology of the host. Therefore, we required more extensive research on *C. albicans* interactome molecules, which directly interact with the host, to better understand the molecular specifics of *C. albicans*-mediated pathophysiology and the altered route by which they counter host defense. For this purpose, a *C. albicans*–human protein–protein interaction (CHPPI) network could assist us in identifying the key virulence-related factors of *C. albicans*. This would help enhance our mechanistic understanding of the putative molecular interactions and functions that *C. albicans* may have to subvert the host during its infection. Except for few studies, host–fungal PPI networks remain virtually unknown. For a fungal disease, identifying virulence factors via the *in vivo* method and thoroughly mapping their host protein interactors take a significant amount of time and effort. In the milieu of this, *in silico* tools to identify possible host targets for fungi are essential. Interolog-based predictions are one of the many computational techniques used to forecast the pathogen–host interaction network (Remmele et al., 2015; Balkenhol et al., 2022; Kataria and Kaundal, 2022).

Here, we elucidated the molecular crosstalk between the host and pathogen protein during oral candidiasis though an integrated network-based approach. We identified three key transcription factors (Rob1p, Tec1p, and Ume6) and their downstream genes (HHT21, CYP5, and KAR2) of *Candida* which hijack host immune response via interaction with crucial module core proteins in a tongue-specific PPIN (tPPIN). We anticipate that analyzing these interactions will enrich our knowledge about the etiology of fungal infections and provide an up-to-date insight into the therapeutic strategies against oral candidiasis.

## 2 Materials and methods

### 2.1 Identification of differentially expressed genes in *C. albicans* and humans during oral candidiasis

Pathogenic genes implicated for oral candidiasis were taken from three research articles (Supplementary Material) (Park et al., 2009; Liu et al., 2015; Lemberg et al., 2022) in which *C. albicans* was cultivated in three oral cell lines. We retrieved 59, 34, and 873 differentially expressed genes (DEGs) of *C. albicans* from the FaDu cell line GSE5340 (Park et al., 2009), OKF6/TRET cell line GS56093 (Liu et al., 2015), and oral keratinocyte cell line (Lemberg et al., 2022), respectively. We prepared an exhaustive list of 948 pathogenic genes (Supplementary Table S1). To obtain host DEGs, we sourced data from two oral squamous cell carcinoma (OSCC) cell lines, namely, HO-N1-1 and HSC, which had been infected with *C. albicans*. These data were extracted from Supplementary Material of GSE169728 (Vadovics et al., 2022). We made an exhaustive list of 6,806 significant human DEGs (Supplementary Table S2).

### 2.2 Reconstruction of the pathogen protein–protein interaction network and gene regulatory network in *C. albicans*

We used STRING v11.5 (Szklarczyk et al., 2019) to build protein–protein interaction networks with the 948 DEGs of *C. albicans*. These networks contain only physical interactions with a cut-off of interaction confidence score ≥ 0.4. The pPPIN was observed to consist of 559 nodes and 4,167 edges (Supplementary Figure S1). We used Cytoscape 3.9.1 (Shannon et al., 2003) to visualize the networks. Furthermore, we also utilized PathoYeastract (Monteiro et al., 2020) databases for predicting TF–target gene (TG) interactions of 559 nodes of the pPPIN under biofilm-forming environmental conditions. The PathoYeastract database contains experimentally validated (RNA-seq—WT vs. TF mutant, microarray WT vs. TF mutant; chip on chip, ChIP) TF–TG association data (Monteiro et al., 2020). For the extraction of TF–TF interaction, we also used the PathoYeastract (Monteiro et al., 2020) database under biofilm-forming conditions. For TG–TG interaction, we used STRING v11.5 (Szklarczyk et al., 2020) with a cut-off of interaction confidence score ≥ 0.4. We obtained a gene regulatory network (GRN) with 9 TFs and 287 TGs (Supplementary Figure S2).

### 2.3 Discerning host–pathogen interaction based on the interolog approach

For constructing a cross-species interaction network, we extracted the whole-genome sequence of *C. albicans* from Ensembl Fungi (Yates et al., 2021) by choosing the Ensembl Fungi Genes 55 database. Since there is a paucity of confirmed experimental data on protein–protein interactions between *C. albicans* and host proteins in the context of oral candidiasis owing to the time-consuming and resource-intensive experimental techniques, we were constrained to utilize a prediction-based technique. Although many studies employed protein structure-based predictions to infer host–pathogen protein interactions (Mariano and Wuchty, 2017), this approach cannot assure the discovery of a significant similarity between proteins from pathogens and those with known structures. Thus, to predict through the best possible way, we chose HPIDB 3.0 (Ammari et al., 2016) that aids the annotation, prediction, and visualization of host–pathogen interactions (HPIs) by retrieving all experimentally established pathogen–host PPI data (Ammari et al., 2016). Moreover, this technique was widely used in various research articles to envision the host–pathogen interaction, e.g., *Leptospira interrogans* and *Homo sapiens* (Kumar et al., 2019), *Burkholderia pseudomallei* and *Homo sapiens* (Loaiza et al., 2020), and *Klebsiella pneumoniae* and *Homo sapiens* (Saha and Kundu, 2021). We initially entered the sequences of *C. albicans* in FASTA format in the HPIDB 3.0. Only those PPIs inferred from experiments such as Y2H, co-immuno-precipitation, and other experimentally robust protocols were considered the template for predicting human–*C. albicans* PPIs. Since false positive interaction is routinely produced using interolog PPI prediction algorithms, to decrease the retention of false positives in our dataset, we applied a two-step procedure. First, we refine the BLAST alignments and generated random sets of sequence identity, e-value, and sequence coverage combinations, i.e., sequence identity (30%, 40%, 50%, and 60%), e-value (1 × 10^−4^, 1 × 10^−5^, 1 × 10^−10^, 1 × 10^−20^, and 1 × 10^−25^), and sequence coverage (40%, 50%, 60%, 80%, and 90%). Next, we chose the combination with sequence identity ≥ 50%, e-value ≤ 1 × 10^−10^, and coverage ≥ 90% for interolog detection as it conferred an optimal number of interactions. Other combinations provided either profuse or trivial interactions which were not suitable for our downstream analysis. Furthermore, we noticed that these screening criteria had also been utilized as the best reciprocal blast hit criteria for deducing human*–Klebsiella pneumoniae* interaction (Saha and Kundu, 2021). Subsequently, considering these stringent criteria, we retrieved 822 interactions, consisting of 412 *C. albicans* and 353 human nodes. Second, we used GO functional similarity enrichment analysis to remove any remaining false positive interolog pairs between the host and pathogen (Saha and Kundu, 2021). We extracted the GO functional annotation of *Homo sapiens* (host) and *C. albicans* from the Ensembl database(Yates et al., 2021) and Ensembl Fungi database (Cunningham et al., 2021), respectively. We classified a pair of homologs to be functionally comparable if they shared at least one GO term. We performed the GO functional similarity enrichment analysis using an in-house perl script and received 119 host proteins interacting with 317 *C. albicans* proteins, constituting 432 interactions (Supplementary Table S3).

### 2.4 Constructing a tongue specific protein-protein interaction network (tPPIN) in humans

For the elucidation of the target network of *C. albicans*, we extracted 3,888 genes expressed in the adult tongue from Expression Atlas (Mabbott et al., 2013) with TPM value ≥ 11. Then, we reconstructed a tPPIN (Supplementary Figure S3) in stringApp in Cytoscape plugin v3.9.1 (Shannon et al., 2003) by considering physical interaction and a cut-off score of interaction confidence score ≥ 0.4.

### 2.5 Identification of core module genes in the sub-tPPIN

We executed a module analysis by using ModuLand (Palotai et al., 2012), Cytoscape plugin v3.9.1 (Assenov et al., 2008). ModuLand helps in determining essential network positions (such as module cores and bridges), physiologically significant groupings, module cores that aid in the identification of biological functions, and inter-modular nodes that play an important role in a range of biological networks (Rodriguez et al., 2020).

### 2.6 Ontology analysis of inferred networks

We performed GO biological function and KEGG pathway enrichment analyses of the DEGs pPPIN and GRN of *C. albicans* using YeastEnrichr (Kuleshov et al., 2016) (Supplementary Table S4). GO biological function and Reactome pathway enrichment analyses for human proteins were performed by using Enrichr (Xie et al., 2021) (Supplementary Table S5). YeastEnrichr and Enrichr are web-based enrichment analysis applications that provide a complete range of functional and pathway annotation tools to assist researchers in comprehending the biological importance of lengthy gene lists (Kuleshov et al., 2016; Xie et al., 2021). We considered those GO biological and pathway enrichment terms having adj. *p* value < 0.05.

### 2.7 Graphical plots

The graphical plots were prepared using Cytoscape 3.9.1 (Shannon et al., 2003; Assenov et al., 2008) and R v4.2.2.

## 3 Results

### 3.1 Reconstruction of a pathogenic network of *C. albicans* during oral candidiasis

In the oral cavity of healthy humans, *C. albicans* is commonly established as a normal commensal. The host immune system continuously tracks the growth rate of opportunistic pathogens. Under stress conditions, they acclimatize themselves by avoiding host immunological challenges by forming biofilm layers, changing their morphology, i.e., yeast to hyphal, which is considered a pathogenic form as well as rapidly growing filamentous form, assisting the progression of pathogenesis. To explore the pathogenesis of *C. albicans* during oral candidiasis, we extracted DEGs of *C. albicans* from three literature supplementary datasets in which *C. albicans* has infected the FaDu cell line (GSE5340) (Park et al., 2009), OKF6/TRET cell line (GSE56093) (Liu et al., 2015), and oral keratinocyte cell line TR146 (Lemberg et al., 2022). We prepared an exhaustive list of 948 DEGs by taking adj. *p* value < 0.05 as a cut-off (Supplementary Table S1). Furthermore, to deduce the relevance of the DEGs extracted from oral cell lines in our study, we employed gene ontology enrichment analysis by YeastEnrichr and found that “cellular response to oxidative stress” (GO:0034599), “cellular response to glucose starvation” (GO:0042149), and “glutathione metabolism”-like functions were enriched (Supplementary Table S4). It was demonstrated that glucose starvation induces the expression of pathogenic genes responsible for oral candidiasis to counter the stress (Solis et al., 2023), and in response to oxidative stress induced in the host during oral candidiasis infection, *C. albicans* utilized the glutathione metabolism pathway (Miramón et al., 2014; Naglik et al., 2017). Moreover, among these DEGs, SAP4, SAP5, and SAP6 are proved to be expressed in the oral epithelia during *C. albicans* infection in immunocompromised individuals, such as HIV-positive patients, and absent in commensal vicinity (Naglik et al., 2008). Moreover, SAP5 was also proven to help in colonization, penetration, infection, target E-cadherin (a major protein in epithelial cell junction), and evasion in host tissues (Naglik et al., 2008; Meylani et al., 2021). These lines of evidence dictate the robustness of our dataset for pursuing further downstream analysis.

Next, we reconstructed a pathogenic pPPIN, consisting of 559 nodes and 4,167 edges using the STRING database v11.5 (Supplementary Figure S1) (Szklarczyk et al., 2020). Consequently, using YeastEnrichr (Kuleshov et al., 2016), we performed GO biological function enrichment analysis and observed that “cellular response to oxidative stress” (GO:0034599), “regulation of response to endoplasmic reticulum stress” (GO:1905897), and “response to unfolded protein” (GO:0006986) were enriched in the pPPIN (Supplementary Table S4). In response to oral candidiasis infection, antimicrobial peptides (AMPs) such as LL-37 have been released by the host in the oral cavity to challenge pathogen infection, which creates stress in the endoplasmic reticulum, unfolding of proteins, and affects cell adhesion of *C. albicans* (Tokajuk et al., 2022). In response to endoplasmic reticulum stress, *C. albicans* regulates the cell wall integrity and endoplasmic reticulum (ER) homeostasis (Hsu et al., 2021) through the Sfp1p protein and aids in oral candidiasis. Moreover, we also performed KEGG pathway enrichment analysis and noticed that “autophagy,” “endocytosis,” “arginine biosynthesis,” “pyruvate metabolism,” and “peroxisome” were enriched in the pPPIN interacting proteins (Supplementary Table S4). Previous reports have indicated that pyruvate metabolism serves as a virulence factor in *C. albicans*, contributing to localized tissue ketosis in the host as well as compromising the typical defensive action of the host raised via neutrophil activation during OPC (Saeed, 2000; Huppler et al., 2015; Altmeier et al., 2016), while arginine biosynthesis is implemented by the pathogen for hyphal formation to escape host phagocytosis aiding in eliminating *C. albicans* in the oropharyngeal cavity (Ghosh et al., 2009; Jiménez-López et al., 2013; Černáková and Rodrigues, 2020). Thus, it could be inferred from the functional analyses of the pathogenic protein that they are solely implicated for infection and withstanding the hostile environment in the host.

Now, in response to environmental cues, the transcriptional circuit controls gene expression and its regulation. Therefore, the TFs that regulate the 559 TGs in the pPPIN might play a crucial role in their TG expression under stress conditions. Thus, we constructed the GRN (Supplementary Figure S2), which consists of three types of interactions: TF–TG, TF–TF, and TG–TG. Since it was evidenced that biofilm development plays a crucial role in oral candidiasis (Cho et al., 2021), we searched those TFs which control gene expression during biofilm-forming conditions using the PathoYeastract database. Finally, we constructed a GRN with 300 nodes and 1,232 edges in which 9 TFs control the expression of 287 TGs. Afterward, using YeastEnrichr, we performed GO biological function enrichment analysis of the GRN. We noticed that several stress-responsive functions such as “cellular response to alkaline pH” (GO: 0071469), “cellular response to glucose starvation” (GO: 0042149), “cellular response to starvation” (GO: 0009267), and “positive regulation of transcription from RNA polymerase II promoter in response to stress” (GO: 0036003) were enriched in the GRN (Supplementary Table S4). The functions revealed in our analysis were also found to be relevant in establishing oral candidiasis as it was found that a low oxygen level, decrease in the flow rate of saliva, and pH dysbiosis in the oral cavity help in the expression of the SAP gene in *C. albicans* to thrive in the oral niche and also help in adherence (Schaller et al., 2005; Vila et al., 2020). Moreover, acidic and alkaline pH decrease the expression of cell wall polysaccharides and interaction with sIgA, which aid *C. albicans* to proliferate and evade host response (Bikandi et al., 2000). Thus, the pH-sensing pathway Rim101 of *C. albicans* plays a pivotal role during oropharyngeal candidiasis, and the rim101Δ/Δ mutant significantly reduces the virulence activity of *C. albicans* in a murine model of oropharyngeal candidiasis (Nobile et al., 2008; Mayer et al., 2013).

Concurrently, we performed KEGG pathway enrichment analysis of the GRN and observed that “Glycolysis/Gluconeogenesis,” “Endocytosis,” “Nitrogen metabolism,” and “Autophagy” pathways were enriched (Supplementary Table S4). It was observed in a previous report (Villar and Dongari-Bagtzoglou, 2008) that during oropharyngeal candidiasis, the macrophage helps identify the mannose ligand of *C. albicans* through dectin-1. Endocytosis of the pathogen through the macrophage creates a nutrient starvation condition, which makes them shift from glycolysis to gluconeogenesis, and this nutrient flexibility helps them form hypha and aid in escaping from the macrophage (Mayer et al., 2013). Thus, these function and pathway enrichment analyses clearly depict that the proteins involved in the gene regulatory network of *C. albicans* play a key role in stress-induced pathogenesis during oral candidiasis.

### 3.2 Identification of potential *C. albicans* targets in the human proteome

To detect the *C. albicans–*human cross-species PPI, we created an *in silico* methodology. First, we extracted the whole-genome sequence of *C. albicans* from EnsemblFungi (Yates et al., 2021), and afterward, using an interolog approach, we identified the *C. albicans*–host protein–protein interaction network (CHPPIN) by a rigorous homology search against the host proteome by using the HPIDB 3.0 database (Ammari et al., 2016). We found a putative PPI between 412 *C. albicans* proteins and 353 human proteins constituting 822 PPIs, using a strict reciprocal blast search. Next, we attempted to demonstrate that the *C. albicans* proteins involved in interactions with the human host proteins in the interolog PPI are true homologs of pathogen proteins to mediate potential interactions with the host proteins. For this, we used an *in silico* GO function similarity analysis of the homologous proteins between humans and *C. albicans*, in which we retrieved the functional annotations from the Ensembl genome browser (Cunningham et al., 2021) and Ensembl Fungi (Yates et al., 2021) for humans and *C. albicans,* respectively. We observed that 317 of 412 proteins (∼77%) from *C. albicans* share at least one GO function with the relevant homologs to the human protein. Therefore, it might be concluded that the 317 *C. albicans* homologs that make up the CHPPIN were likely to be enriched in real functional homologs. We mapped 317 *C. albicans* proteins with a putative PPI to identify the human counterpart proteins. As a result, we obtained 317 *C. albicans* homologs interacting with 119 human proteins, constituting 432 PPIs (Supplementary Table S3).

**FIGURE 1:**
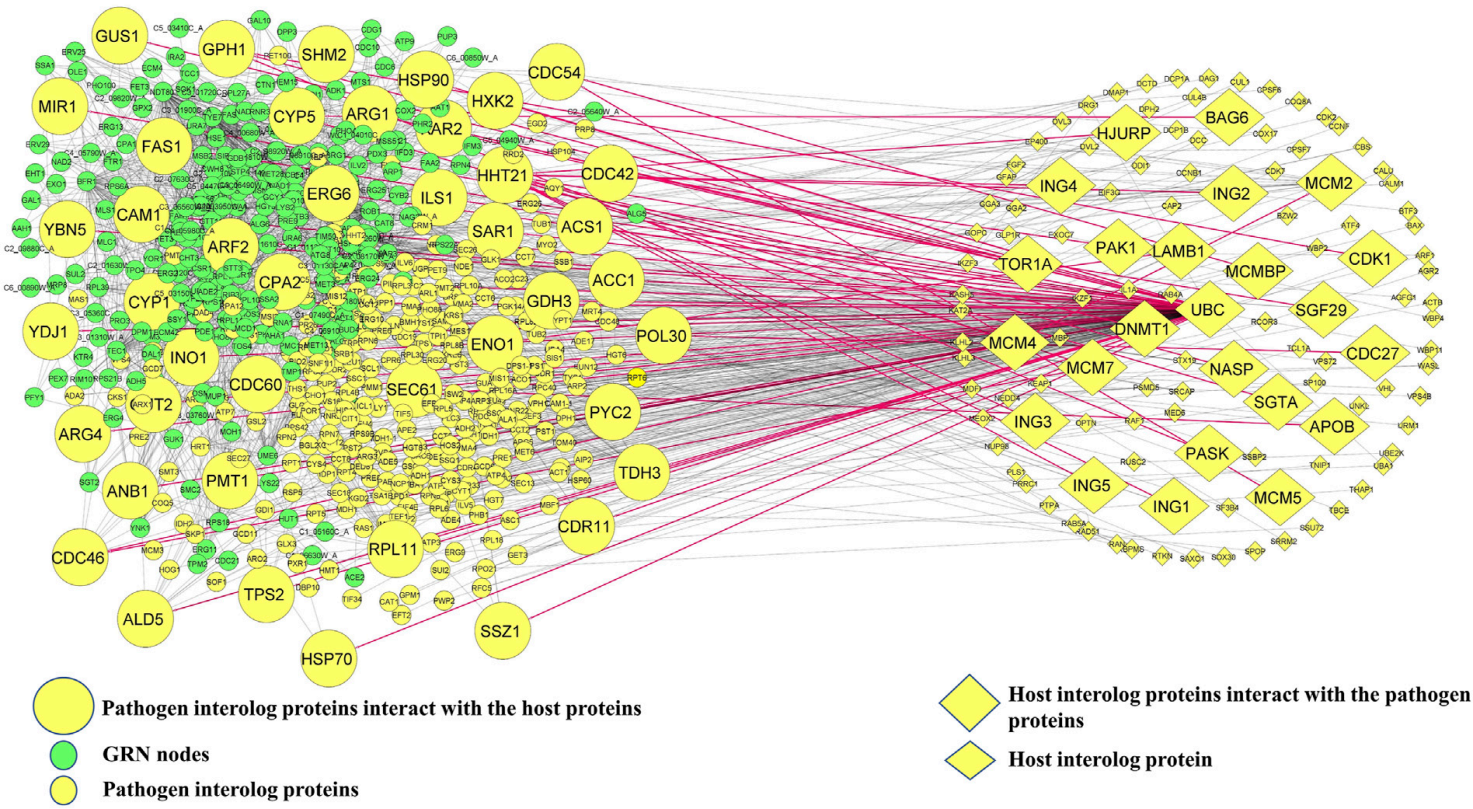
Host–pathogen interaction network. Green ellipsoid nodes represent the GRN, and small yellow ellipsoid and diamond nodes represent pathogenic and host interolog proteins, respectively. Mapping GRN nodes with pathogenic interolog proteins yields 44 *C. albicans* proteins and the corresponding 24 interolog host proteins which are shown in large size. The red solid lines represent 62 edges of direct interaction between host and pathogen proteins.

In the next step of the *in silico* workflow, we attempted to extract those *C. albicans* proteins from the interologs which have the potential to mediate oral candidiasis as previously mentioned. To pursue this, we mapped 317 *C. albicans* interologs with 300 proteins of the GRN implicated in oral candidiasis. We obtained 44 proteins intersecting between these two pools of proteins. Thus, it could be stated that these 44 proteins help *C. albicans* establish a pathogenic repertoire in the oral cavity of the host. We then mapped these *C. albicans* proteins to the interologs to obtain the corresponding human proteins. Meanwhile, we received 24 human proteins which directly interact with 44 *C. albicans* proteins. Thus, it is fascinating to know and explore the molecular crosstalk between 44 *C. albicans* proteins interacting with 24 human proteins, constituting 62 PPIs in the oral mucosa during oral candidiasis (Figure 1).

### 3.3 Reconstruction of the *C. albicans* potential targeted network in the host

**FIGURE 2:**
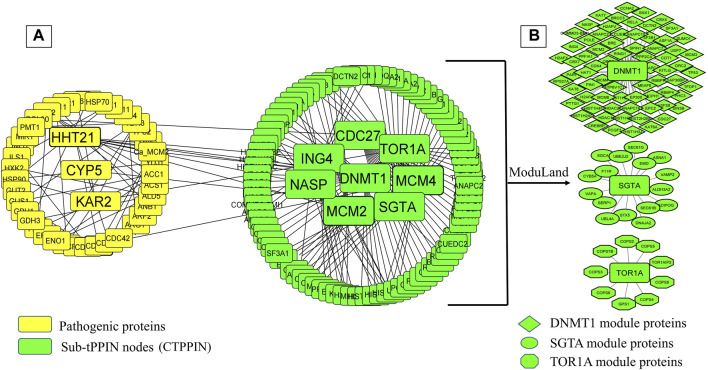
*C. albicans-*targeted protein–protein interaction network (CTPPIN). **(A)** Yellow round rectangular nodes of pathogenic proteins, which directly interact with the tPPIN (green round rectangular nodes) and generate the sub-tPPPIN or CTPPIN with 99 nodes and 119 edges. **(B)** Core modules in the CTPPIN. Green diamond nodes represent the DNMT1 module composed of 72 proteins, green ellipsoid nodes represent the SGTA module composed of 17 proteins, and green octagonal nodes represent the TOR1A module composed of 10 proteins.

**FIGURE 3:**
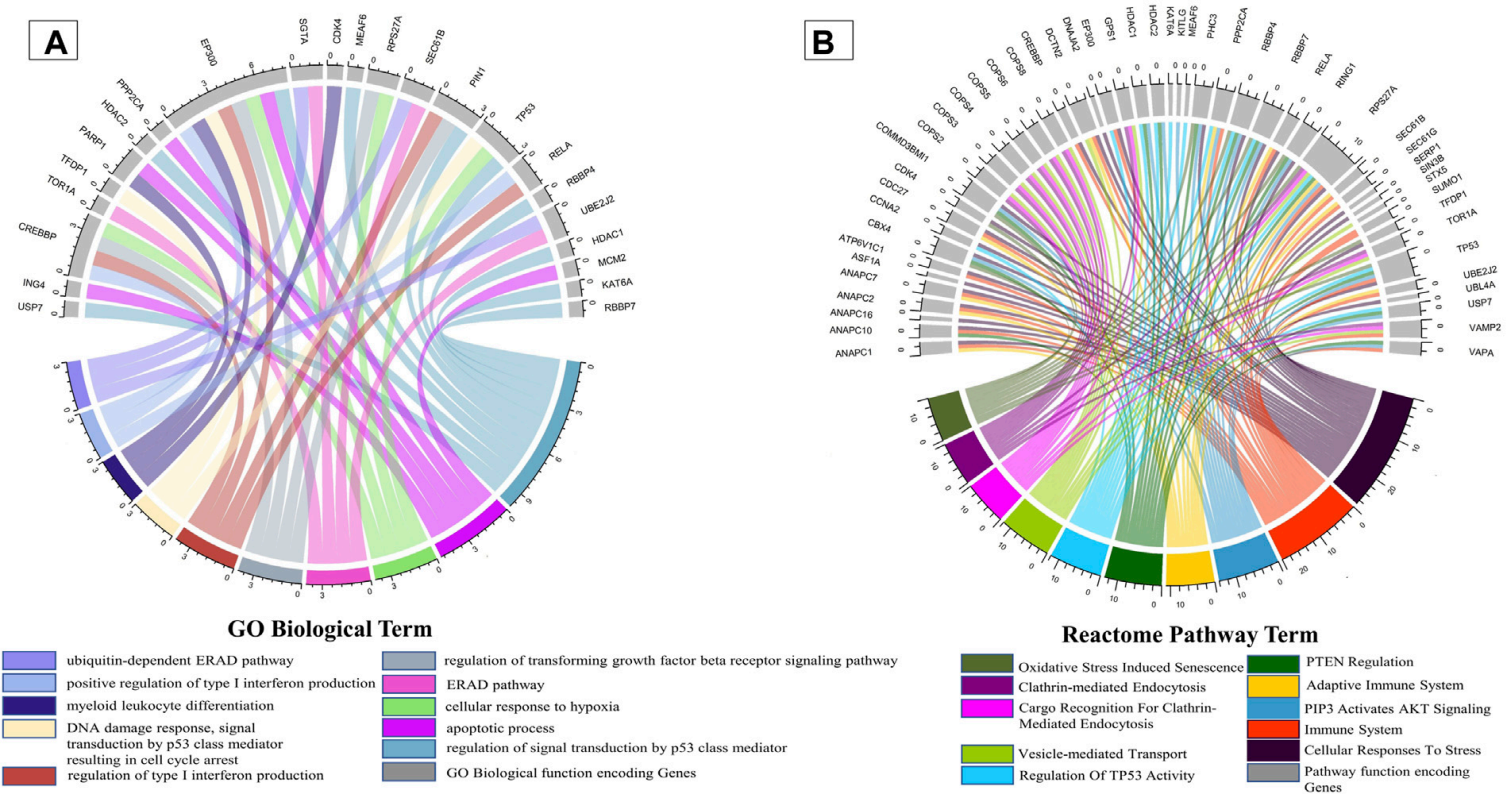
Enrichment of immunological functions and pathways in the CTPPIN. Circos plots representing **(A)** the functions or biological processes and **(B)** Reactome pathways of the protein sub-tPPIN targeted by *Candida*. The length of the ribbons represents the number of host targets which mediate the corresponding function.

It is well known that oral candidiasis involves *C. albicans* infections in the tongue and other oral mucosal regions and is characterized by fungal overgrowth and penetration of surface tissues (Singh et al., 2014; Millsop and Fazel, 2016). Thus, all the 24 human proteins previously mentioned might not be involved in oral candidiasis if they are not expressed in the oral tissues. Thus, we built a tongue-specific PPI network to obtain candidate human proteins in oral candidiasis. First, we extracted 3,888 genes of the tongue with TPM values ≥ 11 (medium expression level) from the Expression Atlas (Mabbott et al., 2013) and then reconstructed the tPPIN comprising 2,775 nodes and 24,889 edges using stringApp in Cytoscape plugin v3.9.1 by considering physical interactors and cut-off score ≥ 0.4 (Assenov et al., 2008) (Supplementary Figure S4). Next, to identify or locate the target network of *C. albicans,* we mapped 2,775 tPPIN nodes with 24 proteins of the host which directly interact with 44 *C. albicans* proteins (Figure 2). We acquired a *C. albicans-*targeted PPIN (CTPPIN), which we can contemplate as a sub-PPI network in the tPPIN, in which we perceived that either of the interacting nodes interacts with *C. albicans* proteins. Since the CTPPIN comprising 99 nodes and 119 edges is tongue specific, it could be inferred that it might be implicated in oral candidiasis (Figure 2). To substantiate this, we checked the underlying biological processes and metabolic pathways of the proteins of the CTPPIN. We performed GO biological function enrichment analysis using Enrichr (Xie et al., 2021). Figure 3 depicts that the sub-tPPIN targeted by *Candida* is enriched with several crucial immunological functions and pathways which confer the potential to the host to resist the pathogen infection. Interestingly, apart from the immunological functions, host cell repair functions such as “nucleotide-excision repair DNA damage recognition” (GO:0000715), “apoptotic process” (GO:0006915), “cellular response to hypoxia” (GO:0071456), “modulation by the host of symbiont process” (GO:0051851), “positive regulation of type I interferon production” (GO:0032481), “DNA Double-Strand Break Response” (R-has-5693606), “PIP3 Activates AKT Signaling” (HASHSA-1257604), “Clathrin-mediated Endocytosis” (HASHSA-8856828), “Class I MHC-Mediated Antigen Processing And Presentation” (HASHSA-983169), and “Toll-Like Receptor 3 (TLR3) Cascade” (HASHSA-168164) were enriched in *Candida-*targeted proteins (Supplementary Table S5). Villar and Zhao (2010) demonstrated that during *C. albicans* infection, apoptosis occurred for the removal of apoptotic bodies containing pathogens by secondary phagocytes (Doran et al., 2020). The occurrence of epithelial damage and the manipulation of macrophage apoptosis create conditions conducive to fungal colonization and infection (Camilli et al., 2021), which corroborates our observation on the involvement of proteins in the *candida-*targeted sub-tPPIN. Thus, it could be stated that the proteins engaged in the CTPPIN accomplish the functions required to combat or provide early symptoms to detect the pathogenesis stage of *C. albicans* in humans.

### 3.4 Explicating the molecular link between the host and pathogen during oral candidiasis

**FIGURE 4:**
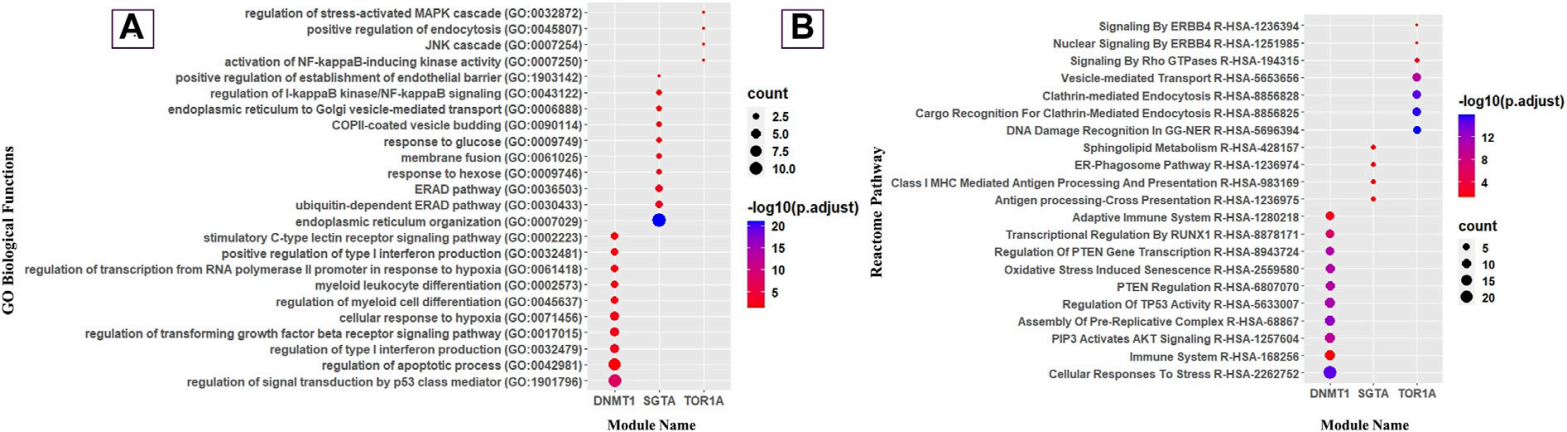
Gene Ontology and Reactome pathway analyses of modules. **(A)** Dot plot of immunological GO biological function and **(B)** immunological reactome pathway enrichment of modules (top 10 GO biological functions and pathways by count).

Deciphering the molecular link between *C. albicans* pathogenic genes and human genes would provide a holistic view to know the exact scenario during oral candidiasis. To identify the key molecular players in the CTPPIN, we performed module analysis using ModuLand (Kovács et al., 2010) in Cytoscape plugin v3.9.1 (Assenov et al., 2008) and obtained three core modules—DNMT1 (consists of 72 proteins), SGTA (consists of 17 proteins), and TOR1A (consists of 10 proteins) (Figure 2). Next, using Enrichr (Xie et al., 2021), we shortlisted among these module core genes which are well-characterized immunological players. We found that the DNMT1 module significantly enriched (p. adjust <0.05) with 24 immunological function and 85 immunological pathway terms (Figure 4; Supplementary Table S5) consists of 46 proteins. Similarly, the SGTA core module was enriched with 34 immune-related function and 4 pathway terms (Figure 4; Supplementary Table S5), encoded by 13 core module proteins, and the TOR1A module was enriched with seven immunological function and four pathway terms (Figure 4; Supplementary Table S5), composed of eight proteins. From the function and pathway enrichment analyses, we could confer that a considerable fraction of proteins, i.e., 63.8 % in the DNMT1 module, 76.4% in the SGTA module, and 80% in the TOR1A module, are implicated for first-line immunological response against the pathogen (Table 1). Thus, dysregulation in the expression of three core module nodes could hamper the host response against *C. albicans* during oral candidiasis. To know the expression of core module nodes during a diseased state, we mapped it with 6,806 DEGs extracted from the oral carcinoma cell lines (HO-1-N1 and HSC) GSE169278 (Vadovics et al., 2022) infected with *C. albicans* (Supplementary Table S2). Subsequently, functional analyses on the DEGs showed the enrichment of “Regulation of Epithelial to Mesenchymal Transition (EMT)” (GO:0010717), “Regulation of Mesenchymal Cell Proliferation” (GO:0010464), “PI3K/AKT Signaling in Cancer” (HASHSA-2219528), “Signaling By WNT in Cancer” (HASHSA-4791275), “Signaling By TGF-beta Receptor Complex In Cancer” (HASHSA-3304351), and “Constitutive Signaling By AKT1 E17K In Cancer” (HASHSA-5674400) (Supplementary Table S5). It was previously reported that infection of *C. albicans* in the HSC-2 cell line upregulates a set of genes associated with EMT, which in turn help in the progression of OSCC (Krisanaprakornkit and Iamaroon, 2012; Jayanthi et al., 2020; Vadovics et al., 2022). Moreover, the PI3K/AKT pathway, Wnt pathway, and TGF-β pathway play a crucial role in the EMT process and could be considered indicators of metastasis (Chen et al., 2008; Krisanaprakornkit and Iamaroon, 2012; Lakshminarayana et al., 2018; Sasahira and Kirita, 2018). From the aforementioned ontology analysis of diseased condition DEGs, we could decipher that they help in the progression of oral cancer when infected with *C. albicans*.

**TABLE 1:** Core module proteins in the host having immunological function and their expression pattern derived from the GSE169278 dataset in the diseased state. Proteins in bold represent deregulated proteins in each module.

Modules	Immunological function-enriched proteins
DNMT1	ORC3, CDT1, ANAPC7, **DCTN2**, **PPP2CA**, **CREBBP**, **RELA**, **HDAC2**, **RPS27A**, **PARP1**, TFDP1, **EP300**, **USP7**, **RBBP4**, **CCNA2**, **CBX4**, **SIN3B**, CDK4, SUMO1, DNMT1, ANAPC16, ATP6V1C1, CDC27, HDAC1, MCM3, RING1, **TP53**, **ASF1A**, **BRCC3**, **KITLG**, MCM4, RBBP7, ANAPC1, ANAPC10, COMMD3-BMI1, MCM6, **MEAF6**, PHC3, **ANAPC2**, POLE, **KAT6A**, **PTTG1**, **MCPH1**, **ING4**, **KAT7**, **PIN1**
SGTA	**SGTA**, F11R, **VAPA**, ADIPOQ, **VAMP2**, **STX5**, **ALDH3A2**, SEC61B, **SEC61G**, UBE2J2, **EMD**, **CYB5A**, **SERP1**
TOR1A	COPS4, **COPS2**, **COPS3**, COPS5, COPS6, **TOR1A**, GPS1, COPS8

Subsequently, after mapping of three core module nodes with diseased condition DEGs, we observed that 54.3% of nodes (*n* = 46) of the DNMT1 module, 69.23% of nodes (*n* = 13) of the SGTA core module, and 37.5% of nodes (*n* = 8) of the TOR1A core module were up- or downregulated in diseased conditions (Table 1). Thus, it could be demonstrated that the deregulation of these crucial network proteins during oral candidiasis facilitates the pathogen to subvert host defense to establish pathogenesis in oral tissues in diseased condition. Now, it would be imperative to examine which of these deregulated nodes of the modules are directly targeted by the pathogen. From the list of potential interologs, we obtained three core module proteins, i.e., ING4, SGTA, and TOR1A, which interact with the HHT21, CYP5, and KAR2 protein of *C. albicans*, respectively. Intriguingly, it is also evident from our DEG analysis (Supplementary Table S1) that these three genes HHT21 (log2FC = 1.80), KAR2 (log2FC = 2.46), and CYP5 (log2FC = 1.37) of *C. albicans* are upregulated during oral candidiasis. Searching for the TFs regulating the expression of these three pathogenic genes from the GRN (Supplementary Figure S2) revealed that HHT21 is tuned by Tec1 and Wor1p, CYP5 is regulated by Rob1p, and KAR2 is controlled by Tye7 and Ume6p. Moreover, we perceived that some of the TFs of these three *C. albicans* genes are up/downregulated during infection (Table 2). Thus, it could be worthwhile to confer that these module-targeting pathogenic proteins, along with their regulators, play significant roles in establishing the infection in the human oral mucosa.

**TABLE 2:** Core module proteins and the expression of their interactors, along with TFs of the pathogen during oral candidiasis, retrieved from Supplementary Material of the study by Lemberg et al. (2022).

Core module protein	*C. albicans* TG	TG expression value (log2FC)	*C. albicans* TF	TF expression value (log2FC)	Association between TFs and TGs
DNMT1 (ING4)	HHT21	1.80	Tec1p	−2.50	**-**
SGTA	CYP5	1.37	Rob1p	−2.25	**-**
TOR1A	KAR2	2.46	Tye7p, Ume6	−1.48, 2.09	**-,+**

## 4 Discussion

This research aimed to determine the virulence mechanisms of *C. albicans* in the host during oral candidiasis as well as to fill the dearth of evidence on interactions between the pathogen and the host proteins during oral candidiasis. To achieve our goal, we first reconstructed a *C. albicans* pPPIN, and to collect information about the regulation of pPPIN nodes under a biofilm-forming condition, we built a GRN which consists of 300 nodes and 1,232 edges and comprises 9 TFs and 287 TGs. Afterward, we built a host (human)–pathogen (*C. albicans*) interaction network (HPIN) using the interolog technique through HPIDB database 3.0 (Ammari et al., 2016), and the inferred interactions were screened. Since false positive interaction is routinely produced using the interolog PPI prediction algorithms, to decrease the retention of false positives in our dataset, we utilized screening criteria, E-value = 10^−10^, query coverage = 90%, and sequence identity > 50% including GO functional enrichment similarity. Following these criteria, we received 317 *C. albicans* proteins which interact with 119 host proteins. Meanwhile, mapping of 317 *C. albicans* proteins with 300 nodes of the GRN yielded 44 *C. albicans* and 24 corresponding host target genes which directly interacted with each other (Figure 1). The interolog approach has traditionally been used to predict a fungus–host PPI (Remmele et al., 2015; Balkenhol et al., 2022). The HPIDB also uses experimentally verified fungus–host PPI data as a template to predict the PPIs. The arduous and time-consuming experimental approaches for inferring PPIs are the reason why there are few experimentally validated fungus–host PPI data. However, the HPIDB approach has several drawbacks, including homology dependence and the inability to forecast protein interactions that are particular to a given strain or lineage. All the molecular targets inferred in this work were computationally predicted, although they still need additional experimental confirmation.

Since oral thrush is produced in the mouth or tongue during oral candidiasis, to understand the impact of candidiasis on the host, it is crucial to figure out the *Candida-*targeted genes which are being expressed in tongue tissue. For this, we mined 3,888 genes of the tongue from the Expression Atlas (Mabbott et al., 2013) and constructed a tPPIN, consisting of 2,775 nodes and 24,889 edges (Supplementary Figure S5). Now, to discern the *C. albicans-*targeted nodes in the tPPIN, we mapped 24 host proteins with 2,775 nodes of the tPPIN. A targeted network (CTPPIN) (Figure 2) of 99 proteins with 119 interactions was obtained in which at least either of the interacting nodes is targeted by the pathogen. Functional analysis of this CTPPIN revealed that it was enriched with crucial stressed-induced responses and immunological function proteins (Figure 3), which indicates their involvement in the host defense mechanism; thus, the targeted network might be subverted by the *C. albicans* proteins to encase its pathogenicity. Consequently, to find out the functional module of the network, we performed a module complex analysis on the targeted network and obtained three core module complexes (DNMT1, SGTA, and TOR1A) (Figure 2). Each complex contained a core protein which could be considered the key protein of the corresponding module. GO biological function and Reactome pathway enrichment analyses of three core module complexes revealed that three modules are engaged in different pathogen clearance mechanisms.

The DNMT1 core module complex consists of 72 proteins, and 63.8% of them are engaged in immunological functions and signaling pathways. We noticed that the “AKT signaling” (R-HSA-1257604) pathway is enriched in the DNMT1 core module, and it is worthwhile to mention that AKT signaling is crucial for T-cell differentiation and it encourages naive CD4 T cells to differentiate into Th17 to prevent oral candidiasis (Tasaki et al., 2018; Abdullah et al., 2021). Moreover, this module was also observed to be involved in the “Regulation of TP53 Activity” (R-HSA-5633007) pathway, which regulates the balance between Treg and Th17 cells. It was previously demonstrated that the depletion of Treg cells causes synchronous depletion of Th17 during oral candidiasis (Pandiyan et al., 2011). Therefore, to inhibit *Candida* infection in the oropharyngeal region, the balance between Treg and Th17 is crucial and binding of TP53 with the Foxp3 promoter helps induce Treg cells (Hua et al., 2020). From this observation (Figure 4), it could be inferred that the DNMT1 module is solely engaged in various T-cell differentiation pathways to raise host immunity against the fungal pathogen.

In our analysis, we also found that the SGTA core module complex consists of 17 proteins, and 76.4% proteins of this core module were significantly enriched with immunological functions and pathways. We detected that they are enriched with the “ERAD pathway (GO:0036503)”-related biological function and “ER–phagosome pathway” (R-HSA-1236974) (Figure 4). Previously, Joffre et al. (2012) mentioned that the ERAD pathway might play an important role in antigen export from endosomes and phagosomes to the cytosol, which could be considered one of the crucial stages for the clearance of *C. albicans* by macrophages in oral candidiasis under an immunocompromised condition (Goupil et al., 2009; Domingues et al., 2022). Disruptions in the process of the ER–Golgi biogenesis and pathway have an impact on antigen presentation and the effectiveness of immune responses to tackle oropharyngeal candidiasis infection. Consequently, it is quite acceptable that the ER is vulnerable to exploitation by certain pathogens (Roy et al., 2006). Moreover, this module was found to be enriched with “endoplasmic reticulum organization” (GO:0007029), “endoplasmic reticulum to cytosol transport” (GO:1903513), “endoplasmic reticulum to Golgi vesicle-mediated transport” (GO:0006888), “protein insertion into the ER membrane” (GO:0051205), and “Class I MHC-Mediated Antigen Processing and Presentation” (R-HSA-983169)-related biological function and pathways (Figure 4). A well-known fact of antigen presentation is that class I-MHC molecules bind peptides generated by proteasomal proteolysis, and they bind them in the ER after the peptides are translocated from the cytosol. Subsequently, during OPC in individuals with immunocompromised conditions, such as HIV, the buccal epithelium serves as a place where cytotoxic T-lymphocyte responses, specifically driven by MHC class I-restricted CD8^+^ T cells, are initiated. Importantly, these responses occur independent of assistance from CD4^+^ cells (Desvignes et al., 1998). Thus, SGTA core module proteins are exclusively involved in the “class-I MHC-mediated antigen processing and presentation” (R-HSA-983169) pathway to elicit host adaptive immunity against the pathogens.

Meanwhile, the TOR1A core module complex consists of 10 proteins, and 80% of them play immunological roles. Subsequently, it was traced that they are mostly involved in the “positive regulation of endocytosis” (GO:0045807) biological function and “Clathrin-mediated Endocytosis” (R-HSA-8856828) pathway (Figure 4), which is a prerequisite step for the internalization and identification of the hyphal form of *C. albicans* during oral candidiasis (Swidergall and Filler, 2017), and in response to that, the host activates the NF-κB, mTOR, and Akt pathway to prevent cellular damage (Moreno-Ruiz et al., 2009; Moyes et al., 2015; Netea et al., 2015). Thus, unlike the other two modules, TOR1A module proteins are basically involved in pathogen internalization and subsequent excitation of effector molecules.

When function and pathway enrichment analyses confirmed the importance of module proteins in host defense, we were curious to investigate their expression patterns in a diseased state, which could strengthen our proposition. Since it is known that oropharyngeal candidiasis is common in cancer patients, we checked the expression of core module proteins by mapping them with an exhaustive list of 6,806 DEGs, which were extracted from the human oral carcinoma cell line infected with *C. albicans* GSE169278 (Vadovics et al., 2022), and discerned a substantial amount of immunological players from the three modules which are deregulated during disease states (Table 1). Deregulation of the nodes in the modules hampers the active communication with their neighbor nodes, which ultimately restraint their functionality.

Now, deregulation of these nodes in the pathogen-targeted network could be succeeded by the direct interaction of the pathogen or via their interacting nodes which are the targets of the pathogen. We were interested to find out the deregulated nodes that are directly targeted by the pathogen and retrieved three pathogen proteins, i.e., HHT21, CYP5, and KAR2, which were significantly expressed during oral candidiasis and found to be directly interacting with core module nodes ING4, SGTA, and TOR1A, respectively. SGTA and TOR1A are the main key proteins of their corresponding module with which all other proteins are connected (Figure 2). Thus, expression deregulation of these two proteins followed by the pathogen target could pose a severe threat to the host. Though DNMT1 is the key protein of the respective module, we did not find any direct association of this protein with *Candida* proteins; rather, we attained a direct interaction with ING4, which is also a hub protein (*n* = 10) in the DNMT1 module (Figure 2). Thus, targeting this protein would inevitably hinder the functionality of the module.

In subsequent research, these three pathogenic proteins HHT21, CYP5, and KAR2 were also evidenced to play a vital role in the pathogenesis of *C. albicans* during oral candidiasis. In the chromatin-level biofilm formation gene circuit, it has been observed that HHT21 acts as a crucial marker for biofilm formation (Rai et al., 2019). Likewise, CYP5 interacts with macrophages in response to stress and pathogenic conditions (Fernández-Arenas et al., 2007; Hirakawa et al., 2015), and in a previous report, KAR2 has been evidenced to help in the secretory pathway (translocation of protein into the ER) and play a pivotal role for *C. albicans* transition from commensal to a pathogenic organism (Morrow et al., 2011). Moreover, KAR2 was reported to be alleviated during the disruption of retrograde protein trafficking, biofilm formation, and antifungal drug resistance conditions (Liu et al., 2014; Li et al., 2015). Biofilm formation is one of the key attributes to identify the oral candidiasis condition (Dongari-Bagtzoglou et al., 2009; Furletti et al., 2011; Muadcheingka and Tantivitayakul, 2015).

Next, in order to find out the regulatory genes of these three pathogen proteins, we investigated the GRN of *C. albicans* and observed that Tec1p and Wor1p act as regulators of HHT21, while Tye7p and Ume6 control the expression of KAR2 and Rob1p regulates CYP5. Now, the expression pattern of these TFs and their corresponding target genes in the pathogen during oral candidiasis would provide the clue to establish their involvement in disease pathogenesis. Investigating the DEGs given in Supplementary Data of the study by Lemberg et al. (2022) (Supplementary Table S1) provided some intriguing results. The transcription factor Tye7p is downregulated (log2FC = −1.48) and Ume6p is upregulated (log2FC = 2.09), while its target gene KAR2 is upregulated (log2FC = 2.46), which is also affirmed in previous studies which showed that Tye7p is negatively and Ume6p is positively associated with the KAR2 protein (Bonhomme et al., 2011; Martin et al., 2011). On the other hand, Rob1p is downregulated (log2FC = −2.25), whereas its target gene CYP5 is upregulated (log2FC = 1.37), and their negative association is also supported by a previous study (Nobile et al., 2012). Another protein HHT21 was observed to be upregulated (log2FC = 1.80), while its TF Tec1p was downregulated (log2FC = −2.50). Although it was evidenced that HHT21 and Tec1p are positively associated (Lin et al., 2013), we found a negative association between them. Since from our analysis, the expression of Rob1p, Tye7p, and Ume6p with their corresponding target genes is consistent with previous experimental evidence, they could be considered candidate TFs for triggering fungal pathogenesis during oral candidiasis.

Furthermore, these three TFs are substantiated to be a regulator of biofilm formation; e.g., Rob1p is considered a core candidate of biofilm formation and designated as a master regulator (Nobile et al., 2012; Glazier et al., 2017; Rodriguez et al., 2020). Furthermore, Rob1p mutation reduces virulence during oropharyngeal candidiasis (Solis et al., 2022). Ume6p also plays vital roles in the development and establishment of biofilms (Park et al., 2021). Moreover, the role of Tye7p in the pathogenesis of oral candidiasis is yet to be elucidated experimentally. However, there is substantial evidence of its role in different mucosal colonization, i.e., gastrointestinal colonization and hyphal formation, adaptation to metabolic stress (Cheng et al., 2013; Alonso-Roman et al., 2022), and hypoxic condition (Bonhomme et al., 2011). Thus, it could be emphasized that Rob1p, Tye7p, and Ume6p and their downstream genes CYP5 and KAR2 play a pivotal role in aiding *C. albicans* to establish a pathogenic repertoire in the oral cavity to trigger oral candidiasis.

## 5 Conclusion

This is the first report on the detailed molecular map of host–pathogen interaction implicated for oral candidiasis in humans. It provides several lines of evidence that antifungal strategies against *Candida* transcription factors, i.e., Ume6p, Tye7p, and Rob1p, as well as their corresponding downstream genes KAR2 and CYP5, could be adopted to challenge *C. albicans* infection in human oral tissues. However, there is a lack of an experimental dataset on *Candida* infection in oral carcinoma cells; thus, the molecular targets inferred in our study by computational prediction need further experimental validation.

## Data Availability

The original contributions presented in the study are included in the article/Supplementary Material; further inquiries can be directed to the corresponding author.

## Supplementary material

SUPPLEMENTARY FIGURE S1 Pathogen protein–protein interaction network. The pPPIN comprises 559 nodes and 4167 edges.

SUPPLEMENTARY FIGURE S2 Gene regulatory network (GRN). The GRN comprises 9 TF nodes and 287 target genes. Green diamond depicts TFs, and ellipsoid green nodes represent target genes.

SUPPLEMENTARY FIGURE S3 Tongue-specific protein–protein interaction network (tPPIN). The tPPIN comprises 2,775 nodes and 24,889 edges.
